# Good efficacy achieved by telitacicept, corticosteroids and immunosuppressants in the treatment of SLE combined with MOG-AD

**DOI:** 10.1093/rap/rkad088

**Published:** 2023-10-18

**Authors:** Mengxue Tian, Lin Tang

**Affiliations:** Department of Rheumatology and Immunology, Second Affiliated Hospital of Chongqing Medical University, Chongqing, China; Department of Rheumatology and Immunology, Second Affiliated Hospital of Chongqing Medical University, Chongqing, China

Key message

- Telitacicept, corticosteroids and immunosuppressants are effective in the treatment of SLE combined with MOG-AD.

Dear Editor, SLE can infrequently manifest as myelin oligodendrocyte glycoprotein (MOG) antibody disease (MOG-AD) when involving the nervous system [1]. Previous studies have associated MOG antibodies with multiple sclerosis; however, recent research has disputed this correlation, establishing MOG-AD as a distinct disease. MOG is a glycoprotein located on the surface of myelin sheaths, serving as a myelin antigen unique to the CNS. Inflammatory stimulation can lead to CNS inflammation and demyelinating lesions with high mortality and disability rates. Currently, experience in the treatment of MOG-AD is limited and treatment is most commonly based on the strategies used for aquaporin 4 (AQP4)-positive neuromyelitis optica spectrum disorder (AQP4+NMO), including methylprednisolone pulse therapy, IVIG, rituximab and plasma exchange [2]. We report the first case of successful treatment of SLE combined with MOG-AD using telitacicept, corticosteroids and immunosuppressants. However, we note that its mechanism remains unclear.

The patient, a 23-year-old female, was admitted to hospital due to difficulty in urination for 2 weeks, accompanied by symptoms including headaches, unsteady walking and numbness in the limbs and body. She denied joint pain, facial erythema, oedema, vision loss or blurred vision. Physical examination revealed positive Babinski signs bilaterally.

Laboratory tests indicated a high ESR of 26 mm/hour, an anti-dsDNA antibody level of 350 IU/ml, decreased C3 (0.69 g/l) and C4 (0.09 g/l) levels and she was positive for anti-Sm antibody. Routine blood tests and liver and kidney function tests were within normal ranges. Subsequently, the patient underwent brain diffusion-weighted imaging (DWI) and full-spine MRI with contrast. T2-weighted images indicated vascular oedema in the right cerebellar peduncle, pontomedullary junction and medulla oblongata, as well as longitudinal segmental thickening in the cervical, thoracic and lumbar spinal cords, suggesting demyelinating lesions (Fig. 1). In particular, an ‘H’-shaped T2-weighted image enhancement in the central region of the spinal cord was observed (Fig. 1D). Ophthalmologic examination suggested no significant abnormalities in visual acuity, fundus or visual evoked potentials, and optic neuritis was initially ruled out. MOG antibodies were detected at a titre of 1:32, while AQP4, glial fibrillary acidic protein and myelin basic protein were negative, leading to a diagnosis of SLE combined with MOG-AD.

**Figure 1.**
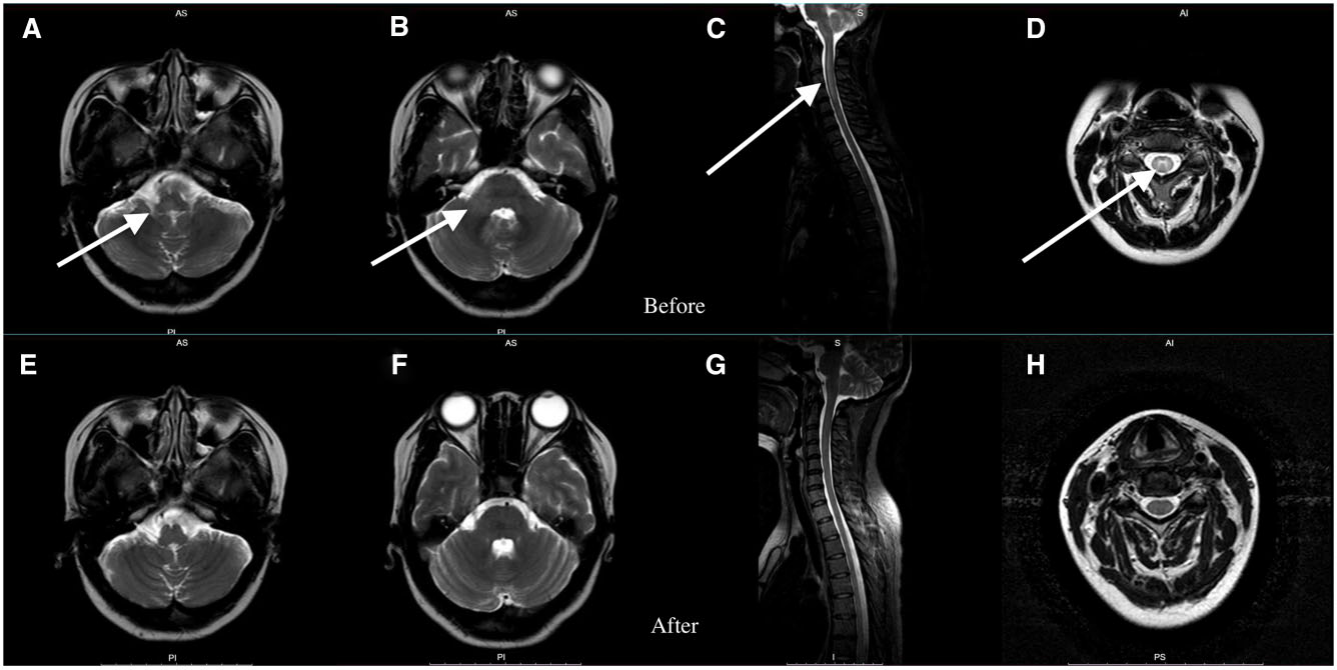
Brain DWIs and full-spine MRI using T2-weighted signal enhancement. **(A)** The medulla oblongata. **(B)** The middle cerebellar peduncle. **(C)** Cervical spinal cord thickening. **(D)** ‘H’-shaped enhancement in the central region of the spinal cord. **(E–H)** The same images after treatment

The patient’s symptoms of headaches and urinary difficulties improved within 2 weeks of treatment using corticosteroids (500 mg/day for 3 days, then 80 mg/day), IVIG, mycophenolate mofetil (750 mg twice a day) and hydroxychloroquine (0.2 g twice a day), and her ESR returned to normal. However, the symptoms of unsteady walking, limb and body numbness and the titre of anti-dsDNA antibodies (348 IU/ml) did not significantly improve. In addition, C3 (0.58 g/l) and C4 (0.04 g/l) continued to steadily decrease.

Considering her financial situation and the drugs’ side effects, the patient refused to use cyclophosphamide, plasma exchange and rituximab. Consequently, telitacicept treatment (160 mg weekly) was added. After 1 week, the patient’s symptoms disappeared completely. ESR, anti-dsDNA and C3/C4 returned to baseline and a repeat brain DWI and full-spine MRI with contrast showed significant improvement (Fig. 1E–H).

In Kavitha Kothur's research [3], MOG antibody-positive patients showed significantly increased expression of the B cell activating factor of the TNF family (BAFF) and a proliferation-inducing ligand (APRIL) in the CSF. These cytokines play an important role in B cell recruitment, clonal selection and amplification [3]. A significant positive correlation between BAFF and APRIL in CSF and soluble markers of activated monocytes and macrophages, sCD163, suggests that BAFF and APRIL levels increase in the CSF during inflammation. This leads to abnormal activation of self-reactive B cells and the production of MOG antibodies [4]. Another experiment demonstrated that anti-BAFF and anti-APRIL antibodies can significantly delay the occurrence and development of autoimmune encephalomyelitis, with anti-BAFF antibodies having a stronger effect, reducing the extent of spinal cord demyelination [5]. Therefore, we speculate that BAFF/APRIL inhibitors might suppress development of the disease. Furthermore, Huntington *et al.* [6] found that the incidence and severity of MOG antibody-related demyelinating lesions were significantly reduced in mice pretreated with hBCMA-Fc. In addition, treatment with hBCMA-Fc after disease onset shortened the course of the disease in mice. At the same time, both of these treatments resulted in decreased MOG antibody titres. These studies indicate that BAFF/APRIL inhibitors have a certain therapeutic effect on MOG-AD.

Telitacicept is a dual inhibitor of BAFF and APRIL, which has been approved by the Chinese National Medical Products Administration for the treatment of SLE in China. Clinical studies on the indications of telitacicept for multiple sclerosis, myasthenia gravis and IgA nephropathy are ongoing [7]. Telitacicept combined with plasma exchange has good efficacy in treating AQP4+NMO patients and is safe and effective in treating experimental autoimmune encephalomyelitis [5, 8]. In this case, we used telitacicept with corticosteroids and immunosuppressants, controlling SLE and rare MOG-AD in this patient. This finding may provide new treatment opportunities.

## Data Availability

All data are incorporated into the article.
